# Transplanted Endothelial Progenitor Cells Improve Ischemia Muscle Regeneration in Mice by Diffusion Tensor MR Imaging

**DOI:** 10.1155/2016/3641401

**Published:** 2016-08-28

**Authors:** Xin-Gui Peng, Yingying Bai, Judy R. James, Darya P. Shlapak, Shenghong Ju

**Affiliations:** ^1^Jiangsu Key Laboratory of Molecular and Functional Imaging, Department of Radiology, Zhongda Hospital, Medical School, Southeast University, Nanjing, China; ^2^Department of Radiology, University of Mississippi Medical Center, Jackson, MS, USA

## Abstract

Endothelial progenitor cells (EPCs) play an important role in repairing ischemia tissues. Diffusion tensor imaging (DTI) was applied to detect the architectural organization of skeletal muscle. This study investigated the feasibility and accuracy of using the DTI to evaluate effectiveness of EPCs treatment. Mouse bone marrow-derived EPCs were isolated, cultured, characterized, and transplanted to hindlimb ischemia mice model. DTI was performed on the hindlimb at postischemia time points. The edema regions of diffusion restriction (high signal in diffusion weighted imaging) were decreased in the ischemic muscle of EPCs treated mice after 14 days compared with the controls. These results from DTI show the lower apparent diffusion coefficient and eigenvalues (*λ*1, *λ*2, and *λ*3) and the higher fractional anisotropy and fiber counts of ischemic muscle on 7 and 14 days after EPCs treatment compared to the controls. There was a significant correlation between fiber counts calculated by DTI and survival fibers evaluated by histological section (*r* = 0.873, *P* < 0.01). Our study demonstrated that the time frame for muscle fiber regeneration after EPCs transplantation was significantly shortened* in vivo*. DTI could be a useful tool for noninvasive evaluation of muscle tissue damage and repair in animal models and patient with ischemic diseases.

## 1. Introduction

There has been evidence of an age-related increase in the lower-extremity peripheral arterial disease in the United States [1]. After ischemia, inflammation, apoptosis, and necrosis happened in local tissue [2, 3], then muscle regeneration was followed by activation of myogenic cells if revascularization can be built [4].

In recent years, there has been growing interest in using stem cells and differentiated progenitor cells as a therapeutic method for the patients with severe ischemic coronary artery or peripheral arterial disease who did not participate in revascularization procedures [5–7]. In 1999, it was first reported that bone marrow-derived endothelial progenitor cells (EPCs) contributed to neovascularization in ischemic tissues [8]. EPCs migrating to ischemic tissue and organs did not always participate in neovessels formation but rather produced a variety of proangiogenic cytokines and growth factors to promote proliferation and migration of preexisting endothelial cells [9–11]. However, the patients with background diseases, such as aging, diabetes, hypercholesterolemia, hypertension, and smoking, may reduce the number and function of circulating/BM EPCs [9]. In our previous study, the cytokines secretion function of diabetic EPCs was significantly decreased compared to normal cell [12]. Stem cell based strategies will expect improving the current therapies.

Recent studies have shown that diffusion tensor imaging (DTI) may detect the architecture of regenerating skeletal muscle or remodeling left ventricle after ischemic injury following EPCs injection [13, 14]. DTI observed that the free diffusion of water in tissue is restricted by membranes and other cellular constituents in physiological or pathological condition, which is orientation dependent for elongated structures, such as nerve fibers and muscle fibers. Orientation dependency was obtained through measuring the diffusion in at least six directions and the diffusion tensor eigenvalues (*λ*1, *λ*2, and *λ*3) can be calculated, which represent the three-dimensional characteristics [15]. Apart from evaluation of the eccentricity of the diffusion ellipsoid by fractional anisotropy (FA), muscle fiber was visualized by fiber tractography. And apparent diffusion coefficient (ADC) displays the size of the diffusing compartment.

In the present study, we focused on two objectives: (1) to examine whether intracardiac transplantation of bone marrow-derived EPCs promotes tissue recovery after hindlimb ischemia and (2) to demonstrate the feasibility and accuracy of* in vivo* DTI in the evaluation of muscle fiber regeneration in a mouse model with hindlimb ischemia.

## 2. Materials and Methods

### 2.1. Isolation and Culture of EPCs

All animal experiments were approved by the Institutional Animal Care and Use Committee of the Medical School of Southeast University (approval ID: SYXK-2010.4987). EPCs were isolated from the tibias and femurs of 4-week-old male C57BLKS/J mice (Shanghai Laboratory Animal Center of the Chinese Academy of Science) as previously described [16]. Six mice were anaesthetized with inhaled isoflurane (1.0–1.5%, KeYuan, Shandong, China) and then killed by cervical dislocation. Aspirated bone marrow was mixed with heparin (100 U/mL, heparin sodium, Shanghai No. 1 Biochemical & Pharmaceutical Co., Ltd., China) in phosphate-buffered saline (PBS, Boster Biological Technology Co., Ltd., China). The mononuclear cell fraction was obtained from a Lymphoprep density gradient (human lymphocytes separation medium, HaoYang Biological Manufacture Co., Tianjin, China) after centrifugation (400 g, 25 minutes; Sigma-Aldrich, St. Louis, USA). The mononuclear cell fraction was collected, washed, and centrifuged. The collected cell pellet was suspended in the growth factor supplemented EBM-2 (Lonza, Switzerland) and plated on fibronectin (Sigma-Aldrich, St. Louis, MO) coated flasks (Corning Inc., NY). After 4 days in culture, nonadherent cells were removed by washing with PBS one time. The culture was maintained through days 7–10. Spindle-shaped cells were observed after 4 days. EPCs were collected for further experiments after 15 days.

### 2.2. EPCs Phenotype Assessment

EPCs were primarily characterized by the use of phase contrast microscopy to evaluate morphology. To assess the endothelial phenotype of EPC colonies, the cells were incubated with acLDL-Dil (Invitrogen Corporation, Carlsbad, CA) diluted at 25 mg/mL concentration in a culture medium for 4 h at 37°C. Lectin binding was analyzed using fluorescein isothiocyanate- (FITC-) conjugated UEA-1-lectin (Invitrogen Corporation). Cells were then examined under a fluorescence microscope (Zeiss, Germany). Immunocytochemistry was used to analyze the expression of various progenitor and endothelial lineage markers [17–19], including CD34 (Santa Cruz Biotechnology Inc., Santa Cruz, CA), CD133 (Abnova Corporation, Taiwan), and CXCR4 (Abcam Biochemical, Cambridge, UK). Cell nuclei were stained with 4′,6-diamidino-2-phenylindole (DAPI, Beyotime Institute of Biotechnology, Haimen, China). Alexa 488-labeled secondary antibodies were examined with confocal microscopy (Olympus, LEXT, Japan). The cell immunofluorescence of CD34, CD133, and CXCR4 was repeated three times, respectively, and averaged among all specimens to measure the positive ratio of the marker. Flow cytometry was performed using a fluorescence activated cell sorter caliber instrument (Becton Dickinson, San Jose, CA) as described previously [18]. The rat monoclonal antibodies, PE-CD34 (BD), fluorescein isothiocyanate-CD133 (eBioscience), PE-VEGF (BD), and PE-CD31 (BD), were used to stain for mouse hematopoietic stem cell and endothelial markers.

### 2.3. Mouse Model of Hindlimb Ischemia and EPCs Transplantation

Athymic nude male mice (Shanghai Laboratory Animal Center of the Chinese Academy of Science) at the age of 5–7 weeks and weighing 15–20 g were anesthetized with inhaled isoflurane (1.0–1.5%, KeYuan). The right femoral artery was exposed and excised with an electrocoagulator (GD350-S3, Hutong Co., Ltd., Shanghai, China) from the proximal origin of the femoral artery to the bifurcation into the saphenous and popliteal arteries [6]. After surgery, micro-CT (MCT-1108, Junhe Co., Ltd., Suzhou, China) was used to record the vascular condition and to evaluate this model by an intracardiac injection of barium sulfate (80% w/v, Meisheng, Fujian, China).

After being anesthetized with isoflurane (1%), forty-eight mice were randomly assigned to a blinded intracardiac delivery of control saline (150 *μ*L each), 1 × 10^6^ cultured EPCs (150 *μ*L each) after 24 hours of surgery.

#### 2.3.1. MRI Scans

All MR experiments were carried out using a 7.0 T small animal magnetic resonance system (Bruker PharmaScan, Ettlingen, ParaVision 5.1 software, Germany). For* in vivo* MR acquisition, anesthesia was induced by inhalation of a mixture of air and 3% isoflurane and maintained by a mixture of oxygen containing 0.5% to 1% isoflurane, respectively. The mouse was positioned supine inside the surface coil and placed in the scanner and monitored using a small animal instrument monitor.

**Figure 1:**
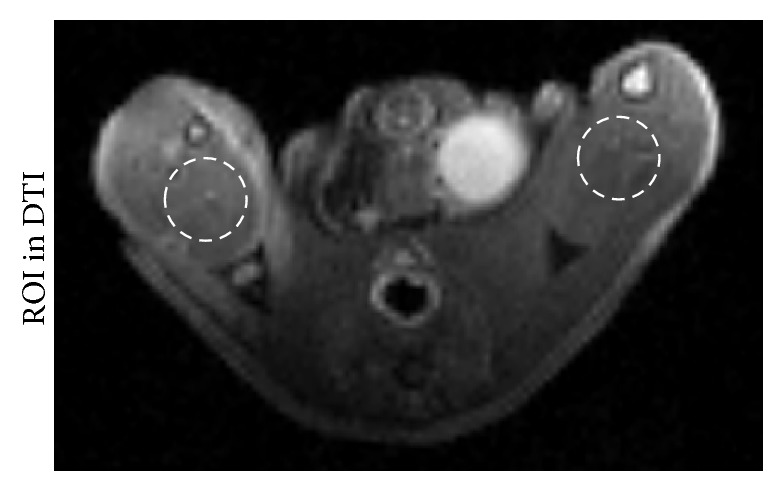
The example of regions of interest (ROI) for FA, ADC, *λ*1, *λ*2, and *λ*3 value of the gastrocnemius. ROI was drawn manually at the ischemic gastrocnemius muscle in DTI maps to measure the FA, ADC, *λ*1, *λ*2, and *λ*3 maps, respectively.

Diffusion weighted images (DWI) of the ischemic hindlimb indicated the presence of edema in the ischemic muscle. The diffusion weighted images were performed at 1, 3, 7, 14, 21, and 28 days after ischemia (*n* = 5 per time point) to study the change in ischemic damage using an echo planar imaging sequence with parameters of repetition time/echo time of 2500/30 ms,* b*-value of 500 s/mm^2^, matrix of 128 × 128, field of view of 3.5 cm × 3.5 cm, slice thickness of 1 mm, and a number of excitations of 1. The area of the edema (white signal in DWI) was measured using Image J software (National Institutes of Health, Bethesda, USA).* In vivo* DTI was performed at a time point according to the following experimental procedure. A spin echo sequence with bipolar diffusion gradients was used to acquire 10 different diffusion directions and five reference images (repetition time/echo time of 5000/28 ms,* b*-value of 0 and 500 s/mm^2^, bandwidth of 250000 Hz at the same FOV, and slice locations of DWI) for a total acquisition time of 5 min. The pixel intensities of the diffusion tensor imaging data set were fitted by using the *b* matrix to obtain the diffusion tensor maps. All imaging gradients were taken into account in the calculation of the *b* matrix [20]. The MR indices were calculated pixel by pixel and averaged for a circular region of interest of 150–180 pixels (6-7 mm^2^) positioned with the gastrocnemius muscle (Figure 1). From the tensor maps, eigenvalues (*λ*1, *λ*2, and *λ*3), mean ADC, and FA were calculated using the software of ParaVision 5.1 (Bruker PharmaScan MRI), where ADC = trace/3 and

(1) $$\begin{array}{c} FA=\frac{\sqrt{{\left(\lambda 1-\lambda 2\right)}^{2}+{\left(\lambda 1-\lambda 2\right)}^{2}+{\left(\lambda 1-\lambda 2\right)}^{2}}}{\sqrt{2\left({\lambda 1}^{2}+{\lambda 2}^{2}+{\lambda 3}^{2}\right)}}. \end{array}$$

Fiber tracking was performed using a software program (DTI Studio, version 2). The FA threshold was set at 0.1 to avoid missing fibers in regions of low FA, and the angular threshold was set low at 20°, at which the fiber tracking stopped if the fiber orientation changed by greater than 20° in adjacent voxel locations in the track. The area of the edema in gastrocnemius muscle was selected to measure the fiber count.

#### 2.3.2. Histological Analysis

To detect the EPCs muscle recovery effect after ischemic injury, the two groups of mice were sacrificed immediately with a mixture of air and 5.0% isoflurane after* in vivo* analysis at days 1, 3, 7, 14, 21, and 28 after ischemia (*n* = 3-4 per time point). Each mouse was perfused transcardially with phosphate-buffered saline, followed by freshly prepared 4% paraformaldehyde (Reagent No. 1 Factory of Shanghai Chemical Reagent Co., Ltd., China) in 0.1 M phosphate buffer (pH 7.4). Gastrocnemius muscles were fixed, dehydrated, embedded, and transversely sectioned into 5 *μ*m pieces (six sections per sample) for hematoxylin and eosin (H&E, Boster Biological Technology Co., Ltd., China) to detect muscle recovery after ischemia injury and Masson's staining (Shanghai Hongqiao Lexiang Medical Reagent Technology Co., Ltd., China) was used to examine muscle fibers. The general rule in Masson's staining is that the less porous tissues are colored by the smallest molecule dye; whenever a dye of large molecular size is able to penetrate, it always does so at the expense of the smaller molecule. The normal muscle cells have small pores and the red dye is able to penetrate, while the red dye is pulled out of the collagen because of the quite porous structure. After ischemia, the larger pores of the injured muscle cell lead to the disappearance of the red color. To examine the difference in the ischemic muscle fiber recovery between the two groups, we calculated the area of red color as survival muscle fiber in Masson's staining. The region of red staining was performed with a histological semiquantitative procedure developed with the MATLAB software (The MathWorks, Natick, Mass.). The artificial areas, such as blood vessels, were manually excluded by a pathologist. Five random microscopic fields were captured in Masson's stained sections of each mouse in each group. The percent of survival muscle fiber was calculated by the following formula, averaged among all fields from a mouse:

(2) Survival  muscle  fiber  %=100×the  area  of  survival  muscle  fiberthe  total  tissue  area.

We measured the average value of these five random microscopic fields per sample (mouse).

Microvessel densities of muscle were calculated from serial sections stained with anti-mouse CD31 (Santa Cruz Biotechnology Inc., Santa Cruz, CA) antibody after being treated for 7 days. CD31 positive vessels were counted from five different fields within each mouse and expressed as the average number of microvessels per square millimeter (mean ± SD) [18].

Hindlimb salvage after ischemia was evaluated after treatment. The outcomes of the treatments for ischemic hindlimbs were measured by three parameters, that is, hindlimb salvage, foot necrosis, and autoamputation, on day 28 after the treatment. The percentages of those measurements were used to compare and evaluate the treatment responses from mice receiving treatment of the control saline and EPCs.

### 2.4. Statistical Analysis

All statistical analyses were performed using SPSS software (SPSS for Windows, version 11.0, 2001; SPSS, Chicago, IL). Numerical data were reported as mean values ± standard deviation (SD). Statistical significance was evaluated with an independent-sample* t*-test for comparison between 2 groups or by ANOVA for multiple comparisons. The Bonferroni procedure was used to correct for multiple comparisons. For statistical comparisons of the relation between the fiber count calculated by DTI and the survival fiber evaluated by Masson's staining, a correlation test was applied. A *P* value of less than 0.05 was considered to indicate a statistically significant difference.

## 3. Results

### 3.1. Characteristics of EPCs

**Figure 2:**
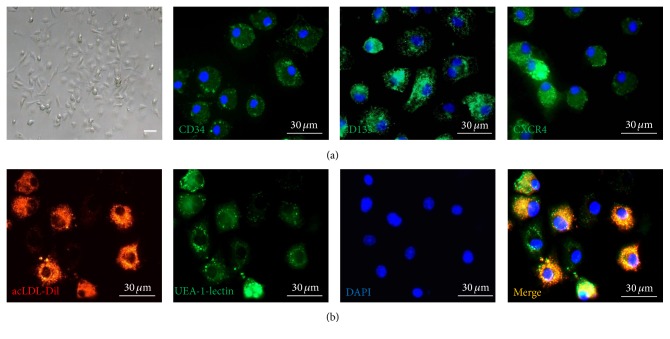
Morphological changes and immunocytochemical analysis in mouse bone marrow-derived EPCs. (a) MNCs changed from globe-like shape to being thin and flat and then round and fusiform at day 7 (bar = 30 *μ*m). These cells were positive by indirect immunofluorescent staining for the markers of hematopoietic stem cells and progenitor cells, CD34, CD133, and CXCR4 (bar = 30 *μ*m). (b) The EPCs were able to take up Dil-labeled acetylated low-density lipoprotein (LDL) and bind the endothelial-specific lectin FITC-labeled lectin after 14 days in culture, which were colocalized in >95% cell (bar = 30 *μ*m).

**Figure 3:**
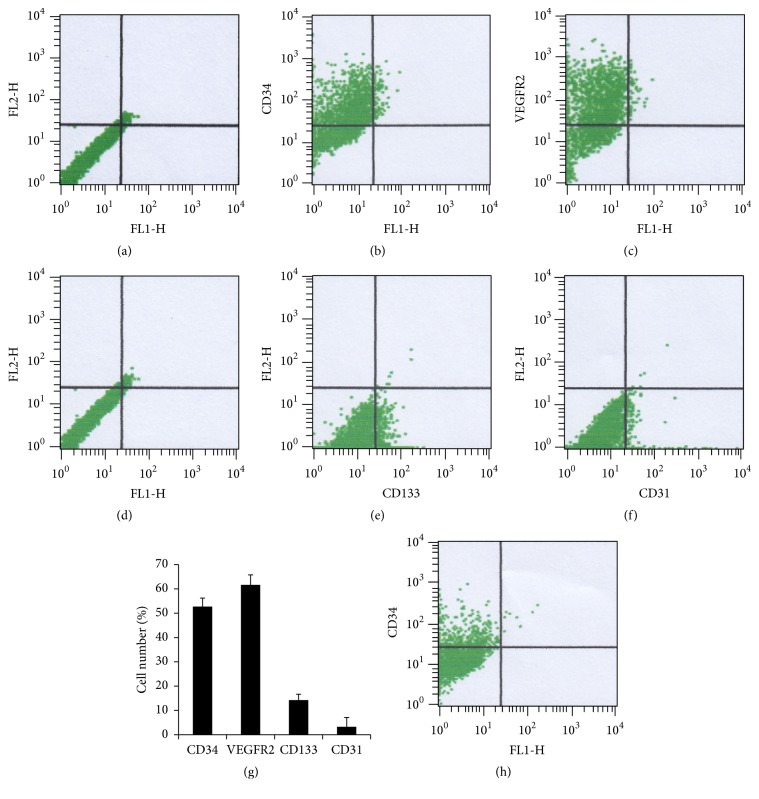
In FACS analyses, EPCs cultured for 15 days were positive for CD34 ((b), 52.73%), VEGFR2 ((c), 61.63%), CD133 ((e), 14.28%), and the mature endothelial-specific marker CD31 ((f), 3.26%). Isotype controls were used in the FACS analyses ((a), (d)). A bar graph (g) showed that the positive rate of CD34, VEGFR2, CD133, and CD31. After being cultured for 30 days, the EPCs were positive for CD34 ((h), 15.65%).

Bone marrow-derived mononuclear cells were isolated and cultured for 7 days. These cells' shape changed from globe-like to spindle-like one (Figure 2(a)). These cells were stained positively by indirect immunofluorescent staining for the markers of hematopoietic stem cells and progenitor cells, CD34, CD133, and CXCR4 (Figure 2(a)). Further examination showed the positive ratio of CD34, CD133, and CXCR4 was about 81%, 76%, and 68%, respectively. Endothelial cell phenotype was characterized by assessing acLDL-Dil uptake and FITC conjugated UEA-1-lectin binding (Figure 2(b)). The ratio of double positive was about 81%. The cells were, therefore, confirmed as bone marrow-derived EPCs. The results of flow cytometry showed the expression levels of CD34 (52.73%), CD133 (14.28%), and VEGF receptor 2 (61.63%) (Figures 3(a)–3(g)). These cells also expressed CD31 (3.26%), which is a specific marker of endothelial cell. After being cultured 30 days, CD34 positive rate of cells deceased to 15.65% (Figure 3(h)).

### 3.2. Evaluation of Hindlimb Ischemia Model

**Figure 4:**
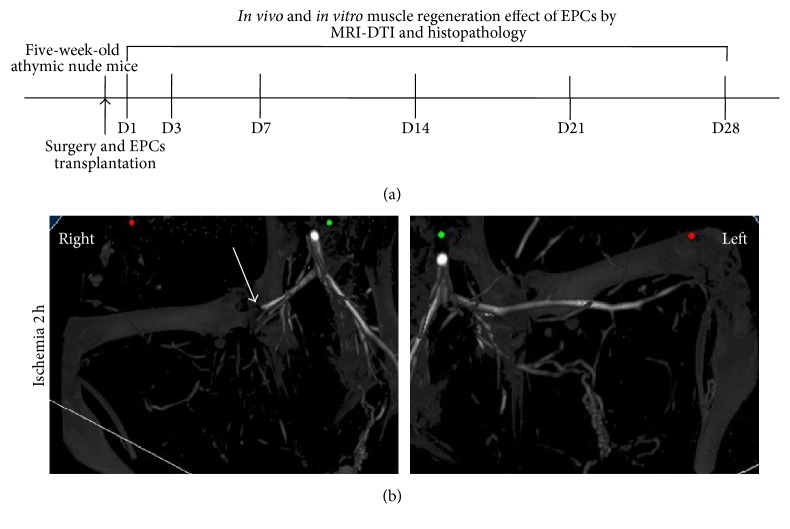
Diagram of the EPCs tracking and treatment evaluation methods (a); representative imaging of micro-CT (b) performed after development of hindlimb ischemia 2 hour. The proximal origin of the right femoral artery was terminated (arrow).

Figure 4(a) was the diagram of the study and treatment methods. Immediately after surgery, the skin color of the right hindlimb turned pale compared with the left normal limb. Micro-CT was performed to detect the dead end in the proximal origin of the right femoral artery (Figure 4(b)).

### 3.3. Muscle Regeneration in the Ischemic Hindlimb after EPCs Treatment

#### 3.3.1. DWI and DTI* In Vivo*

**Figure 5:**
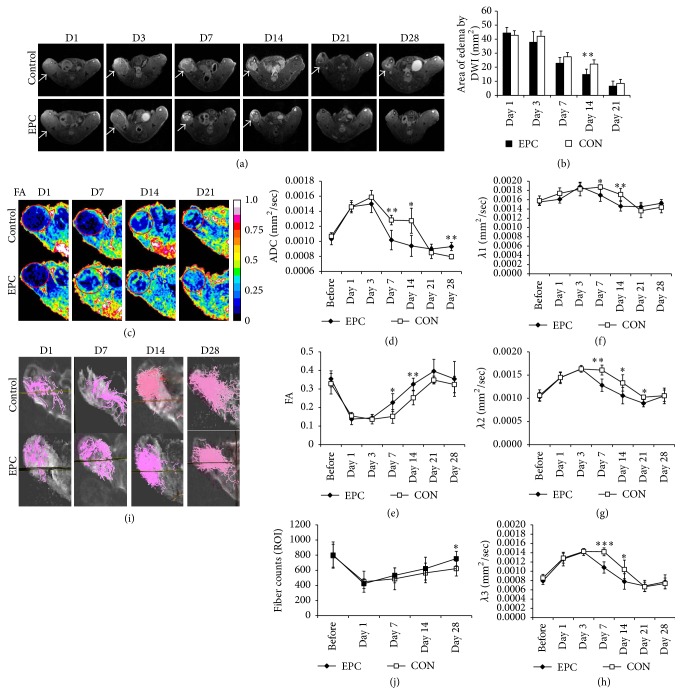
*In vivo* ischemic muscle regeneration effect of EPCs by MR-DTI. (a): Representative DWI at 1, 3, 7, 21, and 28 days in ischemic gastrocnemius muscles in control media injected and EPCs transplanted mice (high signal in DWI indicated diffusion restriction, white arrowhead). A bar graph (b) showed that administration of EPCs significantly reduced the region of edema at day 14 after EPCs transplantation. (c) Representative pixel maps of FA at 1, 7, 14, and 21 days (red circle). Line graphs showed that ADC (d), FA (e), *λ*1 (f), *λ*2 (g), and *λ*3 (h) of EPCs transplantation improved better than that of control group at days 7 and 14 after ischemia. (i) Representative fiber tracking maps at 1, 3, 7, and 28 days. The fiber counts (j) in ischemic gastrocnemius muscles at day 28 after EPCs transplantation were more than those of control group. ^*∗*^ *P* < 0.05, ^*∗∗*^ *P* < 0.01, and ^*∗∗∗*^ *P* < 0.001.

Higher signal in diffusion weighted images of the ischemic hindlimb indicated the edema area of diffusion restriction in the ischemic muscle. The regions of edema in the ischemic muscle at day 14 after treatment with EPCs were smaller than that of control group (Figures 5(a) and 5(b)). At days 1 and 3 after treatment, the ADC in the ischemic muscle increased and the FA value decreased, but there was no difference between the EPCs treated and the control group. However, on days 7 and 14 after treatment, there were significant differences between the EPCs treated and the control group. The ADC values of the ischemic hindlimbs in the EPCs transplantation group were lower than in the control group and the FA values of the ischemic muscle in the EPCs treated mice were higher than that of the control mice (Figures 5(c)–5(e)). Immediately after ischemia, *λ*2 and *λ*3 increased. Seven to fourteen days after treatment, *λ*1, *λ*2, and *λ*3 of the ischemia muscle in the EPCs group were lower compared to the control group (Figures 5(f)–5(h)). In addition, the fiber count of ischemic muscles treated by EPCs was higher than those treated with saline at 28 days (Figures 5(i) and 5(j)).

#### 3.3.2. Masson's Staining

**Figure 6:**
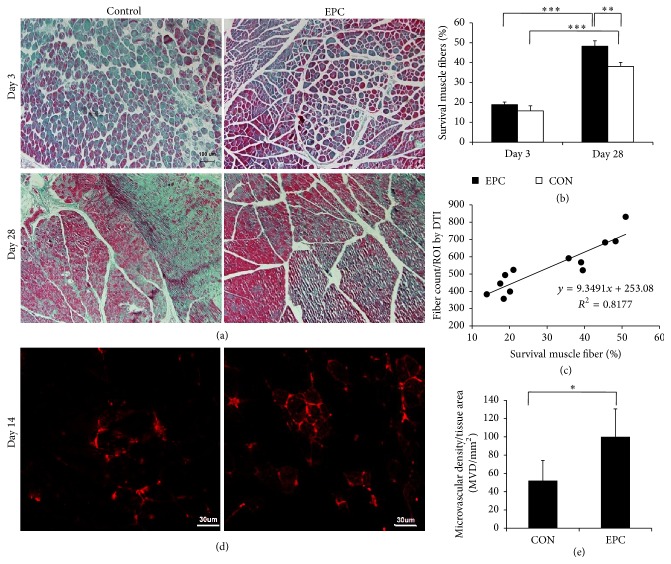
Histologic specimens obtained from ischemia muscle. (a) At days 3 and 28, almost complete tissue recovery using Masson's staining was found in the EPCs treated group. However, in the control group, multiple inflammations and collagen were found (bar = 100 *μ*m). (b) A bar graph showed that the survival muscle fibers of mice treated with EPCs were greater than with the control group. Data is expressed as mean ± SD, ^*∗*^ *P* < 0.05. (c) A line graph showed the correlations between survival muscle fibers by histological method and fiber count measured by DTI (*r* = 0.874, *P* < 0.01). (d) The microvascular density was measured by staining ischemia tissues with CD31 on day 14 after treatment. The EPC treatment augmented vessel density of the ischemic hindlimb (bar = 30 *μ*m). (e) A bar graph showed that the microvascular density (MVD) in the group treated with EPCs was significantly increased compared with that of the control group. ^*∗*^ *P* < 0.05, ^*∗∗*^ *P* < 0.01, and ^*∗∗∗*^ *P* < 0.001.

At day 3 after treatment, there was no difference in survival fiber between the two groups. However, Masson's staining showed that there was a greater expansion of the survival fibers after ischemia at 28 days than at day 3. In the control group, histological examination of muscle sections at day 28 after treatment showed fibrosis areas and numerous necrotic muscular fibers in the ischemic hindlimb (Figures 6(a) and 6(b)). In contrast, in ischemic mice treated with EPCs for 28 days, fibrosis and the number of necrotic muscular fibers were significantly reduced. The percentage of survival muscular fibers in the ischemic mice treated with EPCs was onefold higher than that of control group (Figure 6(b)). In addition, the majority of muscle fibers were intact. There was a significant correlation between fiber counts calculated by DTI and survival fiber evaluated by histopathology (*r* = 0.874, *P* < 0.01) (Figure 6(c)).

#### 3.3.3. Capillary Density Measurement

Immunohistochemical analysis of the ischemic tissue samples with the CD31 marker revealed that the number of capillaries in ischemic muscle from mice treated with EPCs for 14 days was more significant than that of the saline treated control group (*P* < 0.05) (Figures 6(d) and 6(e)).

#### 3.3.4. Tissue Salvage after EPC Treatment

A significant decrease in autoamputation of the ischemic hindlimbs in the EPC treated group was observed after EPC treatment as compared to animals treated with the saline (control group). In the control group, injured limbs were preserved only in 3 of 12 mice (25%), whereas foot necrosis and autoamputation developed in 5 (41.7%) and 4 (33.3%) mice. In contrast, limbs were salvaged in 8 of 15 mice (53.3%) in the EPC treated group. Furthermore, foot necrosis was limited to 4 mice (26.7%), and only three animals (20%) experienced spontaneous limb amputation. The outcomes were significantly different between the EPCs treated group and the control (*P* < 0.01).

## 4. Discussion

In this study, we applied DTI to determine the dynamic changes in tissue repair and muscle fiber regeneration between the EPCs treated group and the control saline group following induced hindlimb ischemia. Furthermore this study also demonstrated that the transplanted bone morrow-derived EPCs improved muscle fiber regeneration.

Chargé and Rudnicki reported that the degeneration and necrosis of the muscle fibers after ischemia result in increased permeability and were accompanied by the activation of inflammatory and myogenic cell [4]. If the collateral circulation could not be built, the incidence rate of limb necrosis, autoamputation, and disability increased significantly [21]. Bone marrow-derived EPCs that contributed to neovascularization in ischemic tissues were reported [9, 22, 23]. In this study, we demonstrated the therapeutic effect of EPCs in the hindlimb ischemia model. The area of bright signal in diffusion imaging after EPCs treatment decreased more quickly compared with control mice* in vivo*, and the numbers of survival fibers were also higher than that of the control mice.

Sotak suggested that the decreased ADC value indicated cell swelling and extracellular diffusion restriction in brain tissue [24]. Compared to brain cell swelling, myocyte swelling resulted in increased ADC value and diffusivity after acute ischemia in skeletal muscle [25, 26]. In our study, we also demonstrated the relationship between ADC and myocyte size, where the areas with swollen myocytes were characterized by higher ADC. The muscle regeneration process is from the outer regions to the inner regions [27]. In our study, treatment with EPCs promoted ischemic muscle recovery with smaller regions of edema in the hindlimb. The ADC value in the EPCs treated group was lower compared to the control group and the area of edema in ischemic muscle was also smaller after 14 days. The results demonstrated that EPCs transplantation promoted tissue recovery* in vivo*. Furthermore, the results of FA, eigenvalues, and fiber counts from DTI suggested that the muscle fibers regeneration after ischemia was better in the transplanted EPCs group than in the control group. Strijkers et al. demonstrated that the increase in three eigenvalues is because of lengthened myocytes on day three after ischemia [28]. The histological findings also indicated a more complete recovery of the muscle fiber in the EPCs treated group versus the control group. In addition, there was a strong relationship between the survival muscle fibers measured by Masson's staining and fiber counts evaluated by DTI.

However, compared to Heemskerk et al.'s report [27], the repair time for ischemic muscles was longer in our study. We found that two factors contributed to this difference. One of the factors was that the induced methods of hindlimb ischemia models were different. In our study, the right femoral artery was excised with an electrocoagulator from the proximal origin of the external iliac artery to the bifurcation into saphenous and popliteal arteries, which induced more severe ischemia compared to ligation. The other factor was that the kind of mouse variety used was different. The wild type mice were used in their study; the nude mice were used in our study. The thymus of nude mice was vestigial, resulting in being incapable of producing mature T-cells [29]. Studies suggest that T-cells play an important role in the development of collateral blood vessels [2, 30]. Therefore, our work indicated that the longer muscle regeneration time required in nude mice may, in part, explain the immune dysfunction that caused the delay in the ischemic muscle fiber recovery.

Brain DTI studies are usually acquired with a* b*-value of 1000 s/mm^2^. However, lower value of 400–600 s/mm^2^ for muscle imaging was recommended because the minimized echo time could maximize the muscle signal (lower T2 relaxation time compared to brain tissue) [31]. Compared to the brain white matter with high anisotropy (FA ≥ 0.7) using approximately 30 gradient directions and the muscle tissue with low FA values (about 0.25), the gradient directions number was about 10 [31, 32]. S. Sinha and U. Sinha demonstrated that there was no significant difference in coefficient of variation from 6- and 13-diffusion gradient acquisitions in human calf muscles at 1.5 T [33].

There were some limitations in this study. First, the MR images were not matched with histologic slices perfectly, so the DTI data did not compare with the histologic detail change. Second, susceptibility artifacts from bone/tissue and air/tissue interface effects on a 7.0 T MR scanner were up to several times greater than those on 1.5 T or 3.0 T scanners, particularly the echo planar based DTI [34]. We used the surface coil and contact closely with the thighs to help decrease the artifacts.

## 5. Conclusion

Our results indicated that the tissue repair and ischemic muscle fiber regeneration after EPCs treatment were significantly better than those of the control group. We demonstrated that the DTI was useful for longitudinally evaluating the regeneration of ischemia muscle fibers after EPCs transplanted treatment in a mouse model with hindlimb ischemia. We propose that DTI can be used for noninvasive evaluation of muscle tissue damage and repair in animal models and patients with ischemic diseases.

## Abbreviations

ADC: Apparent diffusion coefficient
DTI: Diffusion tensor imaging
DWI: Diffusion weighted imaging
EPC: Endothelial progenitor cell
FA: Fractional anisotropy
MRI: Magnetic resonance imaging.
